# The relationship between parental smartphone dependence and elementary students’ internet addiction during the COVID-19 lockdown in China: the mediating role of parent–child conflict and the moderating role of parental roles

**DOI:** 10.3389/fpsyg.2024.1480151

**Published:** 2024-12-11

**Authors:** Chunlan Long, Junjie Liu, Yupan Wu, Siyang Liu

**Affiliations:** ^1^Key Laboratory of Adolescent Cyberpsychology and Behavior (CCNU), Ministry of Education, Wuhan, China; ^2^Key Laboratory of Human Development and Mental Health of Hubei Province, School of Psychology, Central China Normal University, Wuhan, China; ^3^School of Elementary Education, Xiangzhong Normal College For Preschool Education, Shaoyang, China; ^4^No. 2 Complete Primary School of Tangdukou Town, Shaoyang, China; ^5^Siyuan School of Shaoyang Country, Shaoyang, China

**Keywords:** smartphone dependence, internet addiction, COVID-19, parent–child conflict, parental role

## Abstract

During the COVID-19 lockdown in China, the shift of family members’ work and study to online platforms accelerated internet proliferation and led to a growing prominence of internet addiction among younger age groups, posing a threat to individual and societal health development. Previous research has primarily focused on upper-grade elementary students, with relatively less attention given to younger age groups, resulting in insufficient representativeness of the elementary student samples. Additionally, research exploring how parental addictive behaviors are associated with the mechanisms of internet addiction among elementary students has been limited, which affects the development of scientifically based and effective intervention measures for addressing internet addiction in this population. This study, grounded in Family Systems Theory, explores the associative mechanisms between parental smartphone dependence and elementary students’ internet addiction, specifically examining the mediating role of parent–child conflict and the moderating role of parental roles. Using a convenience sampling method, questionnaires were administered to parents from two elementary schools, resulting in 433 valid responses. Structural equation modeling analysis revealed that parental smartphone dependence is associated with elementary students’ internet addiction and that this association is further influenced by the mediating role of parent–child conflict. Additionally, parental roles moderate the relationships between parental smartphone dependence and parent–child conflict, as well as between parent–child conflict and elementary students’ internet addiction. Compared to mothers, fathers’ smartphone dependence is more significantly correlated with parent–child conflict, and conflicts initiated by fathers are more strongly associated with elementary students’ internet addiction. This may be related to China’s traditional “male breadwinner, female homemaker” family culture. Mothers typically assume more parenting responsibilities and establish closer emotional bonds with their children, serving as a protective factor against internet addiction. Therefore, it is recommended that parents reduce smartphone dependence to avoid parent–child conflicts, and that fathers increase their involvement in parenting activities to build stronger emotional connections with their children, thereby fostering healthier internet use behaviors among elementary students. The findings provide valuable insights for developing effective family-based interventions to address internet addiction in children.

## 1 Introduction

The prolonged spread of the COVID-19 pandemic has led to widespread changes in lifestyle, with home isolation becoming the primary response to avoid infection (Bozdağ, 2021). The transition of work and study from offline to online platforms has significantly increased the time parents and children spend with smartphones and internet devices. This shift has also heightened the risk of smartphone dependence and internet addiction among family members. On August 28, 2023, the China Internet Network Information Center (CNNIC) released the 52nd Statistical Report on Internet Development in Beijing, indicating that by June 2023, the number of internet users in China had reached 1.079 billion. Among these, 3.8% are under the age of 10, and 13.9% are aged 10 to 19 (China Internet Network Information Center, 2023). This highlights that elementary students (aged 6 to 12) have become a significant group of internet users. Early internet usage can enhance children’s digital skills and contribute to the high-quality development of the digital society (China Internet Network Information Center, 2023). However, issues of elementary students’ addiction to short videos and online games are also increasingly common (Kawabe et al., 2023). Internet addiction differs from traditional substance addiction; it falls under the category of behavioral addiction. Internet addiction is defined as a phenomenon where individuals exhibit behavior control problems due to prolonged inappropriate internet use, resulting in significant social and psychological impairment (Young, 1998). Internet addiction not only disrupts students’ regular learning but can also positively predict suicidal ideation, posing serious threats to both individual and societal health (Mzumara, 2024; Shen et al., 2020). As elementary students are in a critical period for physical and psychological growth and habit formation, fostering healthy internet use behaviors in this group is of great importance for societal development. Understanding the factors associated with internet addiction among elementary students is crucial for informing the development of effective intervention measures. Previous studies have predominantly focused on internet addiction issues among students in fourth grade and above (Jo and Shin, 2004; Kalkim and Emlek Sert, 2021; Li et al., 2021), with relatively limited research on lower-grade students, resulting in insufficient representativeness for the entire elementary student population. This study encompasses elementary students from all grades and investigates the mechanisms associated with their internet addiction, aiming to address the shortcomings in existing research.

Family Systems Theory posits that an individual’s behavior is closely related to the patterns of interaction within their family, with factors such as parental behavior, parenting styles, and parent–child relationships influencing children’s behavioral habits (Cox and Paley, 1997; Johnson and Ray, 2016). During the COVID-19 lockdown, extended periods of close family interaction provided an opportunity to explore these behavioral associations. The risk of parental smartphone dependence significantly increased during the pandemic lockdown (Ratan et al., 2021). Parental smartphone dependence has been shown to positively associated adolescent smartphone dependence (Gong et al., 2022), and there is a significant correlation between adolescent smartphone dependence and internet addiction (Ayar et al., 2017). However, evidence regarding whether parental smartphone dependence is related to internet addiction among elementary students remains insufficient. Additionally, parental smartphone dependence can disrupt the quality of parent–child interactions (Kildare and Middlemiss, 2017), and family conflict is a known risk factor for children’s internet addiction (Bağatarhan et al., 2023). Whether parental smartphone dependence is related to internet addiction among elementary students through the mediating role of parent–child conflict requires further investigation. Moreover, according to Family Systems Theory (Cox and Paley, 1997), the different roles of parents within the family (father role and mother role) may be associated with varying degrees of correlation with children’s behavior. Although existing research has yet to explore whether parental roles moderate the relationship between parental smartphone dependence and internet addiction among elementary students. Therefore, this study aims to explore the mechanisms by which parental smartphone dependence correlated with internet addiction among elementary students during the COVID-19 lockdown, with a particular focus on the mediating role of parent–child conflict and the moderating role of parental roles, based on Family Systems Theory. This research not only offers theoretical innovation but also provides practical insights for fostering healthy family relationships and online environments, thereby promoting the mutual growth of family members.

## 2 Literature review and research hypotheses

Family Systems Theory emphasizes that the behaviors and actions of family members have reciprocal influences on each other (Cox and Paley, 1997; Johnson and Ray, 2016). For elementary students, observing and imitating family members’ behaviors is one of the key ways they learn. During the COVID-19 lockdown, parents spent extended periods of time with their children, making them important role models for their children’s observational learning. During this period, it has become commonplace for parents to use smartphones for daily activities, work, and entertainment, which can lead to their dependence to smartphones. In psychology, mobile phone dependence is considered a psychological disorder. It is believed that mobile phone dependence is similar to common addictive behaviors, where excessive use of mobile phones for certain reasons leads to physiological or psychological maladjustment in users. Alternatively, it may result from chronic or cyclical obsession caused by repeated use of mobile phones, creating a strong and sustained sense of demand and dependence (Tian and Zhou, 2023). While there is a correlation between smartphone use and the internet, and smartphone dependence shares certain similarities with internet addiction, smartphone dependence is also closely related to its inherent characteristics, such as portability and immediacy. To clarify the differences between the two, existing studies typically discuss smartphone dependence and internet addiction separately (Choi et al., 2015). In China, parents of elementary school students primarily use smartphones, while the students themselves usually do not own smartphones. They often access the internet through other devices, such as computers and smartwatches, in addition to occasionally using their parents’ smartphones. When parents are engrossed in their smartphones, children may become curious about the content their parents are viewing. This curiosity may drive them to access the internet through various devices, leading them to imitate their parents’ behaviors and potentially fall into similar patterns of addiction, ultimately resulting in internet addiction. Numerous studies have shown a significant correlation between parents’ addictive behaviors and those of their children (Arteaga Gómez, 2020). For example, parents’ smartphone dependence has been found to significantly associated with children’s addiction to smart devices (Ayar et al., 2017; Mun and Lee, 2021). Based on this, the study proposes Hypothesis H1: Parents’ smartphone dependence positively correlated with elementary students’ internet addiction.

When parents are absorbed in their smartphones, they often neglect or overlook the people and events around them, which can deteriorate the quality of communication with their children (Gong et al., 2022). Positive parent–child interactions are considered important protective factors against internet addiction (Cai et al., 2021), whereas poor parent–child relationships may lead children to feel rejected, a sensation that is a potential risk factor for internet addiction (Qi et al., 2022). Research by Shayesteh Fard et al. (2016) indicates that children are more likely to become addicted to the internet when their parents exhibit rejection. Conflictual parent–child relationships may result in emotional deprivation in children, driving them to seek psychological compensation in the virtual world. Meta-analysis results also show a significant association between alienated parent–child relationships and a stronger preference for internet use (Zhu et al., 2022), which may lead to internet addiction. Based on this, the study proposes Hypothesis H2: Parents’ smartphone dependence positively correlated with elementary students’ internet addiction through the mediating role of parent–child conflict.

In every family system, different roles fulfill distinct tasks (Cox and Paley, 1997; Johnson and Ray, 2016). In this study, parental roles are categorized into father roles and mother roles based on gender differences. Traditionally, mothers have assumed greater responsibility for caring for their children (Kwok and Shek, 2010). In China, the deeply ingrained “male breadwinner, female homemaker” cultural ideology results in a division of labor in family caregiving. Fathers typically prioritize their careers and provide material support for the family, leading to less communication with their children, while mothers primarily manage the daily life and education of young children. In this cultural context, children often form stronger emotional bonds with their mothers (Leung and Shek, 2016), and such emotional connections are considered protective factors against parent–child conflict and internet addiction (Xu et al., 2024; Zhou et al., 2022). Therefore, compared to fathers, mothers may trigger fewer parent–child conflicts due to their stronger emotional attachment with their children, even when exhibiting smartphone dependence. Although conflicts may arise between mothers and children, the mother’s predominant caregiving role may lessen the child’s sense of emotional deprivation, thereby reducing the risk of internet addiction. Additionally, despite maternal smartphone dependence, as the primary educators of young children, mothers are likely to place greater emphasis on guiding their children away from internet addiction, thus mitigating its occurrence. Based on this, the study proposes the following hypotheses: H3a: Parental role moderates the relationship between parental smartphone dependence and parent–child conflict; H3b: Parental role moderates the relationship between parent–child conflict and elementary students’ internet addiction; H3c: Parental role moderates the relationship between parental smartphone dependence and elementary students’ internet addiction.

**Figure 1:**
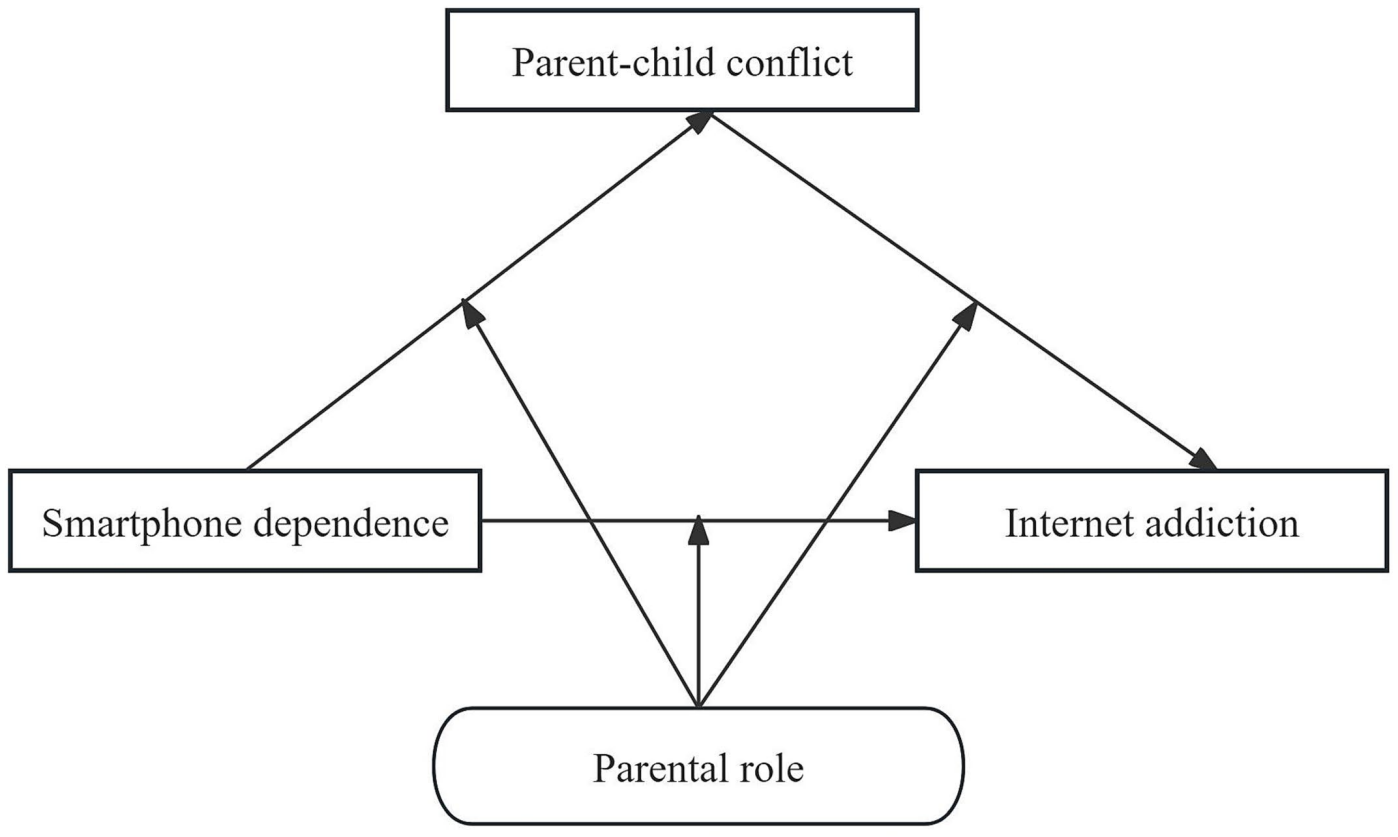
Research hypothesis model.

The proposed research model is illustrated in Figure 1.

## 3 Methods

### 3.1 Participants

The study employed a convenience sampling method, selecting parents from two elementary schools in Shaoyang County, Hunan Province, as the research subjects. Data were collected through electronic questionnaires completed by the parents. A total of 501 questionnaires were collected, with 80 completed by fathers, 353 by mothers (coded as 1 for fathers and 2 for mothers), and the remaining 68 by other family members (such as grandparents and relatives). Based on the research objectives, 433 valid questionnaires were ultimately selected as the effective sample for the study. The detailed demographic characteristics are presented in Table 1.

**Table 1:** Demographic profile of the sample (*N* = 433).

Demographic information	Classification	Frequency (number)	Percentage (%)
Parent roles	Father	80	18.5
	Mather	353	81.5
Child’s gender	Male	234	54
	Female	199	46
Elementary grade	First grade	110	25.4
	Second grade	45	10.4
	Third grade	37	8.5
	Fourth grade	75	17.3
	Fifth grade	71	16.4
	Sixth grade	95	22

### 3.2 Measurement tools

#### 3.2.1 Smartphone dependence

Smartphone dependence was assessed using the Smartphone Application-Based Addiction Scale (SABAS) developed by Csibi et al. (2018). This scale consists of 6 items that measure aspects such as excessive smartphone use, life interference, emotional fluctuations, and withdrawal symptoms (e.g., “I have had conflicts with my family or friends due to my smartphone usage”). The scale uses a Likert-6 point scoring method, where the sum of all item scores quantifies the level of smartphone dependence, with higher scores indicating stronger dependence tendencies. In this study, the Cronbach’s alpha coefficient for this scale was 0.861.

#### 3.2.2 Parent–child conflict

Parent–child conflict was assessed using the Child–Parent Relationship Scale Short-Form developed by Pianta (1992). The original scale includes 15 items that cover two dimensions: close parent–child relationships and conflictual parent–child relationships. This study focused on the conflictual dimension, selecting 7 items (e.g., “I seem to always be struggling to cope with my child”). The scale uses a Likert-5 point scoring method, with parents evaluating their relationship with their child. The total score reflects the level of parent–child conflict, with higher scores indicating more severe conflict. In this study, the Cronbach’s alpha coefficient for this scale was 0.809.

#### 3.2.3 Internet addiction

Internet addiction in elementary students was assessed using the 8-item Diagnostic Questionnaire of Internet Addiction (IAD-DQ) developed by Young (1998), which is based on the DSM-IV criteria for pathological gambling. The original scale includes 8 items and uses a binary response format (yes/no), with a score of 5 or more considered indicative of internet addiction. However, Li et al. (2012) found that the binary response method with a cutoff score of 5 was unreliable. Therefore, this study used a Likert-5 point scoring method for measurement. Considering that younger elementary students may have difficulty understanding and responding to the questionnaire, parents were asked to rate their child’s internet use behavior based on daily observations (e.g., “The child remains engrossed in online activities and feels dissatisfied after stopping”). Higher total scores indicate stronger characteristics of internet addiction in the child. Previous studies have validated the reliability of the method using parental reports of children’s behaviors (Yang et al., 2023). In this study, the Cronbach’s *α* coefficient of the scale was 0.883, indicating good internal consistency.

### 3.3 Survey procedure

Prior to the formal implementation of this study, the research methods were approved by the Ethics Committee of the School of Psychology at Central China Normal University. During the survey process, informed consent was first obtained from the school administrators, class teachers, and parents. The research team then created an electronic questionnaire using Tencent Survey, which was distributed to parents by the class teachers in a parent group. The survey interface provided detailed information about the purpose of the study, the content of the questionnaire, and instructions for completion. It was also emphasized that the questionnaire was anonymous, all data would be used solely for scientific research, and personal privacy would be strictly protected. Parents were able to submit the completed questionnaire directly online. The data collection period was from December 2 to December 24, 2022, coinciding with the pandemic control measures in China.

### 3.4 Statistical analysis

Data organization and statistical analysis for this study were conducted using SPSS 23.0 and the PROCESS 3.5 macro. First, SPSS was used to analyze the distribution of each variable and calculate the correlations between them. Subsequently, PROCESS was employed to perform structural equation modeling.

## 4 Results

### 4.1 Test for common method bias

This study primarily used self-report measures to collect data, which might lead to common method bias. To address this issue, Harman’s single-factor test was conducted using principal component factor analysis on all items of the variables before data analysis. The results indicated that there were seven factors with eigenvalues greater than 1, with the variance explained by the largest factor being 35.63%, which is below the critical value of 40% (Podsakoff et al., 2003). Therefore, it can be concluded that there is no significant common method bias in this study.

### 4.2 Correlation analysis

Table 2 presents the mean scores, standard deviations, and Pearson correlation matrix for the total scores of parents across the variables. The correlation analysis revealed significant positive correlations at the 0.001 level between parental smartphone dependence, parent–child conflict, and elementary students’ internet addiction. Additionally, there are significant correlations between parental roles and smartphone addiction, as well as between parental roles and internet addiction among elementary students.

**Table 2:** The mean, standard deviation, and correlation of research variables.

Variable	M	SD	1	2	3	4
1. Smartphone dependence	2.67	1.14	1			
2. Parent–child conflict	2.41	0.93	0.39^***^	1		
3. Internet addiction	2.11	0.88	0.61^***^	0.47^***^	1	
4. Parental role			−0.12^*^	−0.04	−0.24^***^	1

^*^p < 0.05, ^**^p < 0.01, ^***^p < 0.001.

### 4.3 Test of the mediating effect of parent–child conflict

Following the mediation effect testing procedure, Model 4 was selected in the SPSS macro PROCESS to test the mediation effect. The bias-corrected percentile Bootstrap method was employed with 5,000 resamples, using a two-tailed test to assess the model fit and the significance of the path coefficients. Specifically, parental smartphone dependence was set as the independent variable, elementary students’ internet addiction as the dependent variable, and parent–child conflict as the mediator. The results are presented in Table 3, showing that the 95% confidence intervals did not include zero, indicating that parental smartphone dependence positively correlated with both parent–child conflict and elementary students’ internet addiction. Furthermore, parent–child conflict positively correlated with elementary students’ internet addiction, supporting Hypothesis H1. Additionally, the total effect value of the model was 0.63, with the indirect effect value of 0.11, and the relative effect of the mediation path was 17.5%, supporting Hypothesis H2.

**Table 3:** Testing the mediation model of parent–child conflict.

Independent variable	Dependent variable	*R^2^*	*β*	*t*	95%CI
Smartphone dependence	Parent–child conflict	0.15	0.37	8.78^***^	[0.29, 0.46]
Smartphone dependence	Internet addiction	0.43	0.52	12.77^***^	[0.44, 0.60]
Parent–child conflict			0.29	6.82^***^	[0.21, 0.38]
Indirect effect			0.11		[0.62, 0.16]

^*^p < 0.05, ^**^p < 0.01, ^***^p < 0.001.

### 4.4 Moderation effect test of parental roles

Based on PROCESS Model 59, parental roles were set as the moderating variable, with the child’s grade and gender controlled as covariates. To ensure the reliability of the interaction analysis and reduce the bias caused by unequal sample distribution of parental roles, SPSS was used to standardize the four variables: parental smartphone dependence, parent–child conflict, elementary school students’ internet addiction, and parental roles, converting them into standardized scores. Subsequently, the Bootstrap method was applied with 5,000 resamples to examine the moderating effect of parental roles. The results, as shown in Table 4, indicate that the interaction between parental smartphone dependence and parental roles is significantly negatively correlated with parent–child conflict (*β* = −0.11, *p* < 0.01), but not significantly correlated with elementary students’ internet addiction. This finding suggests that parental roles moderated the relationship between parental smartphone dependence and parent–child conflict, supporting Hypothesis H3a. However, parental roles did not moderate the relationship between smartphone dependence and elementary students’ internet addiction, thus not supporting Hypothesis H3c. Additionally, the study found that the interaction between parent–child conflict and parental roles was significantly negatively correlated with elementary students’ internet addiction (*β* = −0.13, *p* < 0.01), indicating that parental roles moderated the path between parent–child conflict and elementary students’ internet addiction, supporting Hypothesis H3b. The moderating effects under different conditions are shown in Table 5. For the father’s role, the correlation coefficient between smartphone dependence and parent–child conflict is 0.60 (*p* < 0.001); for the mother’s role, the correlation coefficient between smartphone dependence and parent–child conflict is 0.31 (*p* < 0.001). Furthermore, the correlation coefficient between father-induced parent–child conflict and elementary school students’ internet addiction is 0.54, while the correlation coefficient between mother-induced parent–child conflict and elementary school students’ internet addiction is 0.22.

**Table 4:** Testing the moderating effect of parental role.

Independent variable	Parent–child conflict (dependent variable)			Internet addiction (dependent variable)		
	*β*	*t*	95% CI	*β*	*t*	95% CI
Smartphone dependence	0.36	8.01^***^	[0.27,0.45]	0.46	11.79^***^	[0.38,0.53]
Parent–child conflict				0.27	7.06^***^	[0.20,0.35]
Parental role	0.04	0.91	[−0.48;0.13]	−0.16	−4.57^***^	[−0.23, -0.09]
Int1	−0.11	−2.88^**^	[−0.19;-0.04]	0.02	0.46	[−0.06, 0.10]
Int2				−0.13	−2.93^**^	[−0.21, -0.42]
Elementary grade	−0.01	−0.25	[−0.05;0.04]	0.05	−2.83^**^	[0.02, 0.09]
child’s gender	−0.17	−1.89	[−0.34;0.01]	0.18	2.51^*^	[−0.32, -0.04]
R^2^	0.18			0.49		

Int1 = Smartphone dependence * Parental role; Int2 = Parent–child conflict * Parental role ^*^p < 0.05, ^**^p < 0.01, ^***^p < 0.001.

**Table 5:** Effect sizes across different parental roles.

Independent variable	Dependent variable	Parental role	*β*	*t*	95%CI
Smartphone dependence	Parent–child conflict	Father’s role	0.60	7.09^***^	[0.44, 0.77]
		Mother’s role	0.31	5.99^***^	[0.21, 0.41]
Parent–child conflict	Internet addiction	Father’s role	0.54	5.20^***^	[0.34, 0.75]
		Mother’s role	0.22	5.31^***^	[0.21, 0.41]

^*^p < 0.05, ^**^p < 0.01, ^***^p < 0.001.

**Figure 2:**
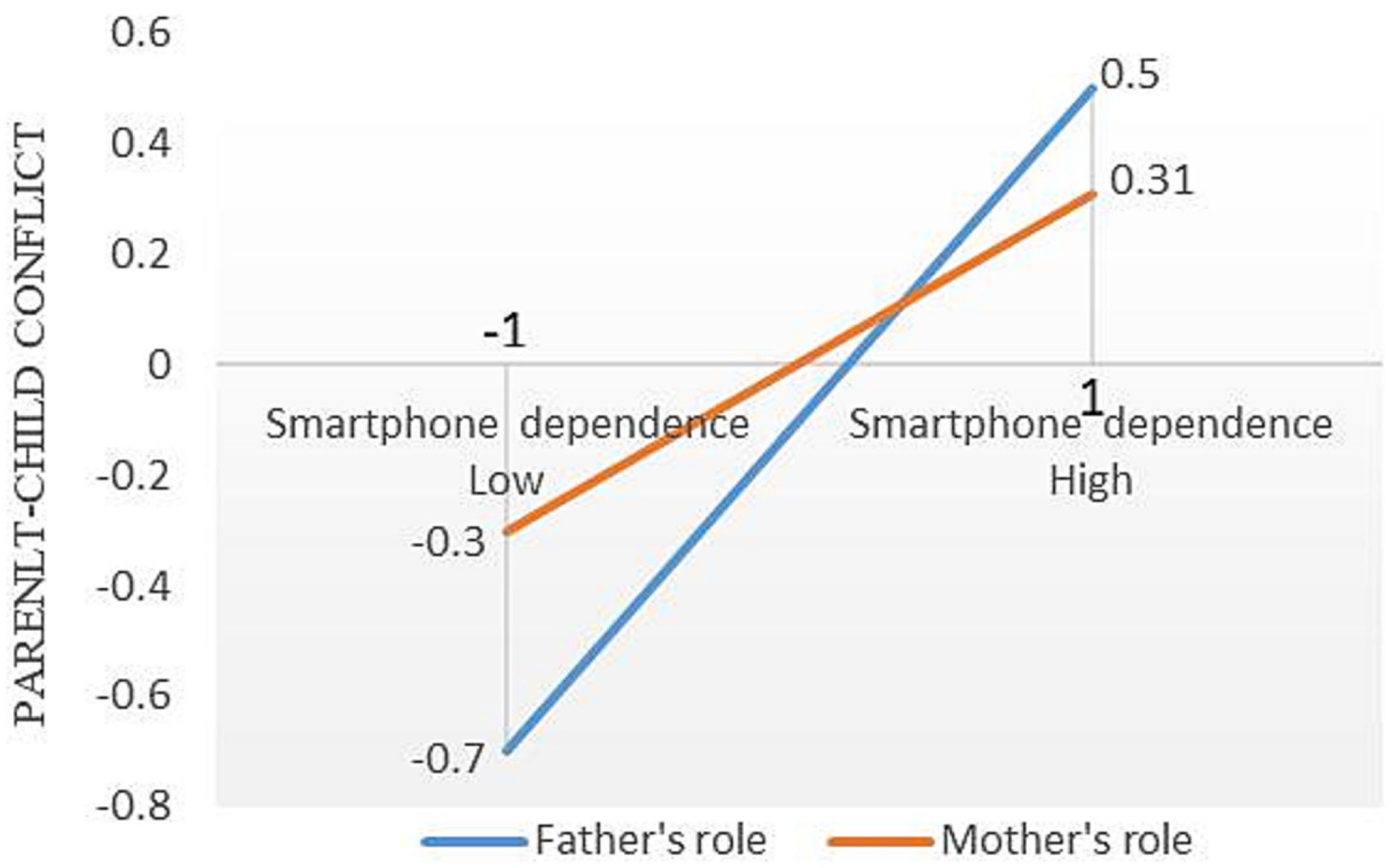
The slope plot of the moderating effect of parental roles on the relationship between parental smartphone dependence and parent–child conflict.

**Figure 3:**
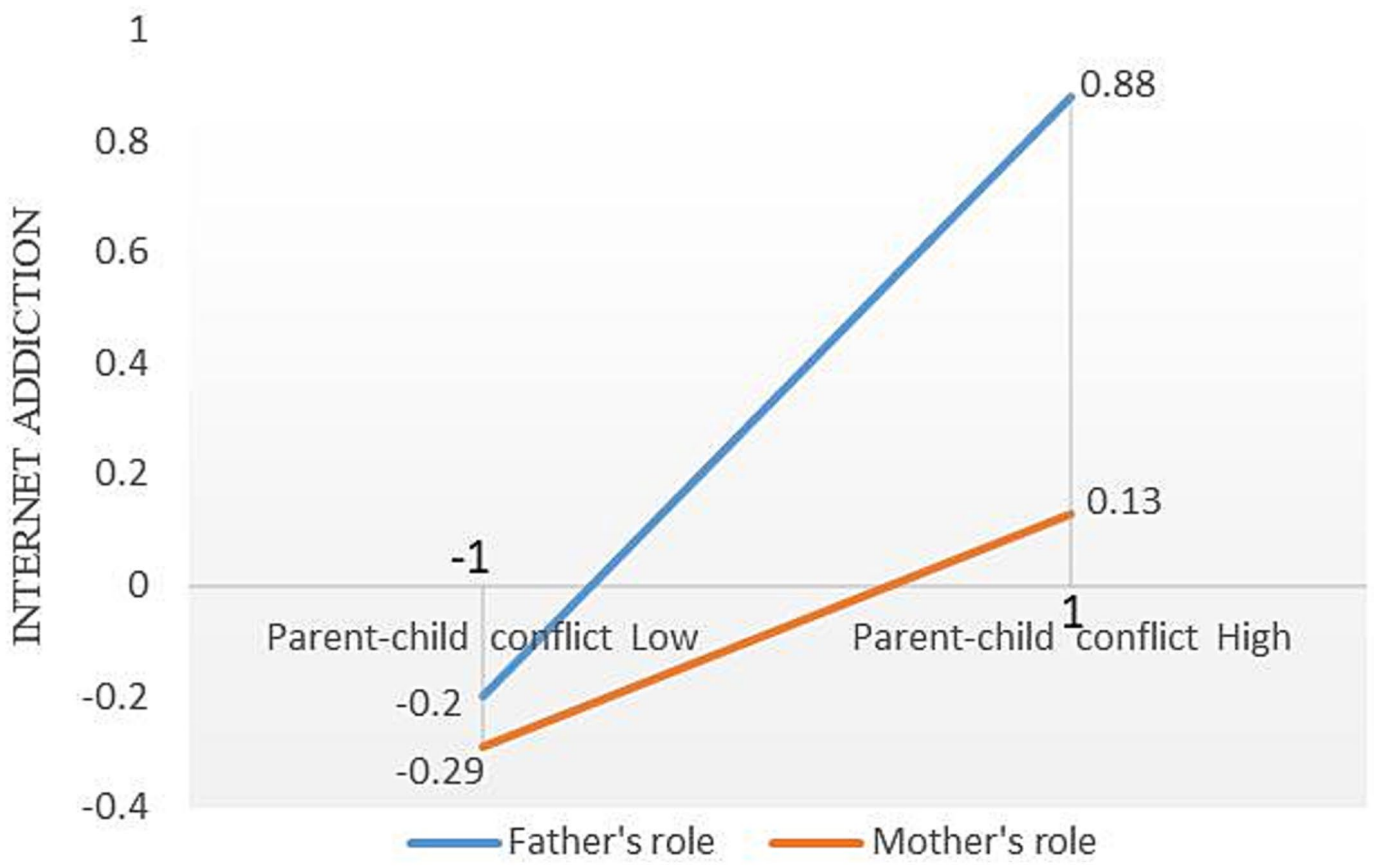
The slope plot of the moderating effect of parental roles on the relationship between parent–child conflict and elementary school students’ internet addiction.

To visually present the moderation effect, simple slope graphs were plotted. Due to the transformation of variables through standardized coefficients, the mean values of parental smartphone dependence, parent–child conflict, and elementary school students’ internet addiction are all 0, with a standard deviation of 1. As shown in Figure 2, when the father’s smartphone dependence is at a low level (M-1SD), the standardized value of parent–child conflict is −0.70; whereas when the father’s smartphone dependence is at a high level (M + 1SD), the standardized value of parent–child conflict increases to 0.50, the slope *k* is 0.6. Similarly, when the mother’s smartphone dependence is at a low level (M-1SD), the standardized value of parent–child conflict is −0.30, and at a high level (M + 1SD), it is 0.31, the slope *k* is 0.305. This suggests that the father’s smartphone dependence is associated with a greater likelihood of parent–child conflict compared to the mother’s. As shown in Figure 3, when the father-induced parent–child conflict is at a low level (M-1SD), the standardized value of elementary school students’ internet addiction is −0.20; when the father-induced parent–child conflict is at a high level (M + 1SD), the standardized value of internet addiction rises to 0.88, the slope *k* is 0.54. In contrast, when the mother-induced parent–child conflict is at a low level (M-1SD), the standardized value of internet addiction is −0.29, and at a high level (M + 1SD), it is 0.13, the slope *k* is 0.21. This further demonstrates that father-induced parent–child conflict is more likely to be related to elementary school students’ internet addiction compared to the mother’s. Furthermore, the index of moderated mediation (Index of Moderated Mediation) is −0.26, with a 95% confidence interval of [−0.42, −0.10], further confirming that parental roles moderate the mediating effect of parental smartphone dependence on elementary school students’ internet addiction through parent–child conflict.

## 5 Discussion

### 5.1 Parental smartphone dependence positively correlated with elementary students’ internet addiction

The study found that parental smartphone dependence directly correlated with elementary students’ internet addiction, with an effect size of 0.50, supporting Hypothesis H1. This result not only validates the Family Systems Theory, which posits that parents’ behaviors significantly associated with their children’s attitudes and behaviors (Cox and Paley, 1997), but also aligns with previous research findings that parental addictive behaviors positively correlated with children’s addictive behaviors (Arteaga Gómez, 2020; Ayar et al., 2017). Particularly during the elementary school years, children are in a critical period for observational learning and habit formation. During the COVID-19 lockdown, with parents and children spending extended time together, parents served as crucial role models in the development of children’s problematic behaviors (Mun and Lee, 2021). Therefore, to prevent internet addiction in elementary students, parents should first reduce their dependence on smartphones to avoid setting a negative example for their children. Engaging in alternative parent–child activities, such as reading together, participating in physical exercises, and playing games, can effectively reduce the time spent on smartphones or the internet within the family, thereby lowering the risk of addictive behaviors in children. These alternative activities not only help strengthen parent–child relationships but also provide a healthy environment for children’s development, further reducing their reliance on the internet.

### 5.2 Parental smartphone dependence positively correlated with elementary students’ internet addiction through parent–child conflict

Here found that parental smartphone dependence can also correlated with elementary students’ internet addiction through parent–child conflict, supporting Hypothesis H2. This result is consistent with previous research, which suggests that parental smartphone dependence associated with the quality of parent–child relationships (Huang et al., 2021; Mun and Lee, 2021), and that the quality of these relationships is closely related to children’s addictive behaviors (Gong et al., 2022; Qi et al., 2022). Parental smartphone dependence not only sets a poor behavioral example but may also lead to neglect of communication with their children as they focus on their devices, failing to meet the emotional needs of the children. This communication barrier may deteriorate the parent–child relationship, potentially leading to conflicts. These conflicts further exacerbate the children’s negative emotions, pushing them to rely more on the online world to compensate for emotional voids, thereby intensifying their internet addiction (Duchesne et al., 2017; Seidman, 2013; Wong et al., 2015). Therefore, parents need to be mindful of their smartphone dependence and should focus on effective communication with their children to establish a healthy parent–child relationship, reducing the risk of internet addiction caused by emotional deprivation. This is particularly crucial in stressful family situations, such as during the COVID-19 lockdowns, when parents and children are confined together at home, potentially heightening anxiety and leading to parent–child conflicts. In such cases, parents should engage in activities that maintain a warm parent–child relationship and alleviate negative emotions, thereby reducing the likelihood of children turning to smartphones or the internet for emotional solace.

### 5.3 The moderating role of parental roles

The study also found that parental roles moderated the pathways between parental smartphone dependence and parent–child conflict, as well as between parent–child conflict and elementary students’ internet addiction, supporting Hypotheses H3a and H3b. This result validates the family Systems Theory’s perspective that different family members, with varying roles and responsibilities, have differential impacts on each other (Cox and Paley, 1997). The study shows that although both parental smartphone dependence and parent–child conflict positively correlated with children’s internet addiction. However, compared to mothers, fathers’ smartphone dependence is more likely to be associated with parent–child conflicts, and father-induced conflicts show a stronger correlation with internet addiction among elementary school students. This finding is innovative. This phenomenon may be related to traditional Chinese family culture. In Chinese families, mothers typically take on the primary responsibility for daily child-rearing, thus they are more attuned to their children’s needs and form close emotional bonds with them. Even if mothers exhibit smartphone dependence, they may still fulfill their child-rearing duties and maintain emotional trust with their children. As a result, even in the presence of parent–child conflict, children may still feel cared for by their mothers, which reduces the likelihood of developing internet dependence. On the other hand, fathers, who may have fewer opportunities to participate in child-rearing, often fail to establish strong, intimate relationships with their children. Therefore, fathers’ smartphone dependence tends to be more closely associated with parent–child conflict, and this conflict is linked to a higher likelihood of children’s internet addiction. This suggests that in family education, it is crucial to encourage both parents to equally share in child-rearing responsibilities and to build close relationships with their children as a protective factor against negative behaviors.

However, the study did not confirm that parental roles moderated the direct pathway between parental smartphone dependence and elementary students’ internet addiction, thus not supporting Hypothesis H3c. This suggests that parental smartphone dependence, whether by the father or the mother, is similarly associated with children’s internet addiction. This finding aligns with previous research, which has shown that parental smartphone dependence significant correlation with children’s addictive behaviors (Gong et al., 2022). Therefore, both parents should be vigilant about their smartphone dependence and avoid setting poor examples for their children.

### 5.4 Significance of the study

Internet addiction poses a significant threat to the healthy development of elementary students. With the widespread use of the internet, internet addiction among elementary students has gradually become one of the major challenges faced by contemporary education. However, systematic discussion and in-depth research targeting this specific group remain relatively scarce. This study focuses on the elementary student population and broadens the sample scope, aiming to gain a more comprehensive understanding of and response to this pressing issue, which holds significant practical implications. Furthermore, compared to schools and society, the family environment exerts a more direct and profound influence on the growth of elementary students. Based on Family Systems Theory, this study explores how parental smartphone dependence behaviors impact elementary students’ internet addiction within the context of prolonged family cohabitation during the COVID-19 lockdown. This research not only tests and enriches the application of Family Systems Theory in the study of internet addiction among elementary students but also provides empirical results that can provide parents with a scientific reference for family education guidance to prevent internet addiction among elementary students. Given the universal nature of the research issues, the findings also hold significant reference value in non-pandemic contexts.

### 5.5 Limitations and future directions

This study has several limitations that need to be addressed. First, the gender distribution of parents in the sample was uneven, with a higher number of mothers participating. This imbalance may have introduced data bias, reflecting characteristics specific to the mother’s role. Future research should aim to increase the sample size of fathers to validate the robustness of the findings. Second, this study primarily employed a cross-sectional design to explore the relationship between parental smartphone dependence and internet addiction among elementary students, which only allows for the establishment of correlations. Future longitudinal research could be conducted to better establish causal relationships. Finally, future studies should explore effective interventions for internet addiction in elementary students from multiple dimensions, including parental smartphone dependence and parent–child conflict. Such research would provide stronger empirical support for scientifically guiding family education and hold significant practical value for broader applications.

## 6 Conclusion

The empirical research revealed that during China’s COVID-19 lockdown, parental smartphone dependence not only positively correlated with internet addiction among elementary students but also this relationship was further associated with parent–child conflict as a mediating factor. Additionally, the parental role moderated the relationships between smartphone dependence and parent–child conflict, as well as between parent–child conflict and elementary students’ internet addiction. Compared to mothers, fathers’ smartphone dependence was more closely associated with parent–child conflict, and such conflict initiated by fathers more significantly linked to internet addiction in elementary students.

## Data Availability

The datasets presented in this study can be found in online repositories. The names of the repository/repositories and accession number(s) can be found in the article/supplementary material.
